# The impact of universal, school based, interventions on help seeking in children and young people: a systematic literature review

**DOI:** 10.1007/s00787-022-02135-y

**Published:** 2023-01-13

**Authors:** Daniel Hayes, Rosie Mansfield, Carla Mason, Joao Santos, Anna Moore, Jan Boehnke, Emma Ashworth, Bettina Moltrecht, Neil Humphrey, Paul Stallard, Praveetha Patalay, Jessica Deighton

**Affiliations:** 1 Evidence Based Practice Unit, Anna Freud National Centre for Children, and Families, London, UK; 2 Social Biobehavioural Research Group, Department of Behavioural Science and Health, University College London, London, UK; 3 Centre for Longitudinal Studies, Social Research Institute, University College London, London, UK; 4 Manchester Institute of Education, University of Manchester, Manchester, UK; 5 School of Health Sciences, University of Dundee, Dundee, UK; 6 School of Psychology, Liverpool John Moores University, Liverpool, UK; 7 Department for Health, University of Bath, Bath, UK; 8 Population Science and Experimental Medicine, MRC Unit for Lifelong Health and Ageing, University College London, London, UK

**Keywords:** Help-seeking, School, Child, Young person, Mental health, Universal

## Abstract

Reviews into universal interventions to improve help seeking in young people focus on specific concepts, such as behaviour, do not differentiate between interpersonal and intrapersonal help seeking, and often report on statistical significance, rather than effect size. The aim of this review was to address the gaps highlighted above, to investigate the impact of universal, school-based interventions on help-seeking in children and young people, as well as to explore longer term impact. Four databases were searched. Data were extracted on country of origin, design, participant, school, and intervention characteristics, the help-seeking concept measured (e.g. knowledge, attitude/intention, behaviour), the duration between baseline and each follow-up (if applicable) and effect sizes at each follow-up. Quality assessment of the studies was undertaken using the Effective Public Health Practice Project (EPHPP) quality assessment tool. Overall, 14 different interventions met inclusion criteria. The majority of the studies were rated low in the quality assessment. Three constructs were most frequently reported a) intrapersonal attitudes towards help-seeking, b) interpersonal attitudes towards help-seeking and c) intrapersonal intended help-seeking. Findings around intervention effect were mixed. There was tentative evidence that interventions impacting interpersonal attitudes produced small effect sizes when measured between 3 and 6 months post intervention and that when effect sizes were initially observed intrapersonal attitudes, this remained at 3–6 month follow-up. Further work should pay attention to implementation factors, understanding the core ingredients needed to deliver effective interventions and whether embedding mental health education could help sustain or top up effect sizes from help-seeking interventions.

**Supplementary Information:**

The online version contains supplementary material available at 10.1007/s00787-022-02135-y.

## Introduction

The prevalence of mental health difficulties among children and young people (CYP) has been increasing in recent years, with one in six CYP aged 5–16 now meeting the criteria for at least one disorder in England in 2020 [1]. Fifty percent of lifetime cases of mental health difficulties have their onset by age 14 [2] making late childhood and early adolescence a crucial period for preventive intervention [3]. When mental health difficulties do emerge, receiving help early can reduce their long-term impact on a range of mental health, physical health, educational and social outcomes [4–6]. However, it is estimated that up to three quarters of young people with diagnosable mental disorders do not, or are unable to, access specialist support [7]. While a number of structural barriers have been identified, including: availability of specialist services, lack of information about specialist services, inflexible services, wait times, complex administrative procedures, costs of services, users’ expectations of providers’ attitudes, and a lack of capacity reported by primary care providers [8]; other reasons include mental health stigma and embarrassment, lack of mental health knowledge, and negative perceptions of help seeking [9].

Exploration into why young people decide to access any mental health support, or not, has also highlighted demographic differences. For example, findings suggest males and older adolescents are more likely to report being concerned about others' reactions [8, 10]. Overcoming these barriers is challenging and early intervention and prevention, such as via school-based programmes, may be beneficial in helping CYP develop the skills needed, so that specialist input is not needed.

### Universal prevention and promotion programmes in schools

Internationally, schools are increasingly responsible for supporting the mental health of CYP [11–13]. Within England, this is evidenced in recent government policies such as the rollout of Mental Health Support Teams in schools as well as mental health and wellbeing modules being incorporated into the Relationships, Sex and Health Education Curriculum [14]. School interventions can be split into universal and targeted (selective or indicated) interventions [15]. Universal prevention addresses whole populations not defined on the basis of risk; selective interventions are targeted at subgroups with an elevated risk of developing a mental disorder; and indicated prevention is targeted at subgroups at high risk and individuals with detectable but subclinical symptoms of a mental disorder [16]. Whether to focus on universal or targeted interventions is debated in the academic literature. Whilst targeted interventions for specific difficulties often yield greater effect sizes than universal programmes [17], they may be more resource intensive by requiring screening and identification of the target population [18] and can also increase both self-stigma and stigma from others due to individuals being singled out [19] which in turn may affect engagement [20]. On top of this, as universal interventions are delivered to all (i.e., a class, school, or local area), rather than working only with at-risk individuals, such programmes can also help tackle the ‘community’-wide factors associated with positive mental health and wellbeing or help-seeking [21]. Moreover, targeted interventions [22] also may not create the desired “immunisation” effect, intervening early to prevent the onset of mental health difficulties through the promotion of adaptive behaviours [23].

When it comes to effect sizes for constructs related to help seeking, including mental health literacy ([MHL; 24], universal interventions appear to fare equally well as targeted interventions [25]. Universal interventions also have the benefit of not singling out those at risk or already experiencing difficulties which can lead to stigma. However, the effect of universal interventions for help-seeking, as opposed to MHL, for young people in schools has yet to be investigated.

### MHL and universal, help-seeking programmes

MHL comprises four broad domains: (1) understanding how to obtain and maintain positive mental health; (2) understanding mental disorders and their treatments; (3) decreasing stigma related to mental disorders, and (4) enhancing help-seeking efficacy (knowing when and where to seek help and developing competencies designed to improve one’s mental health care and self-management capabilities’ [26]. MHL programmes in schools typically focus on educating and changing beliefs and attitudes about mental health difficulties to aid recognition, appropriate support, management, and prevention [27–29]. Low MHL is a significant barrier to seeking help [30].

Help-seeking efficacy refers to knowing when and where to seek help and developing competencies designed to improve one’s mental health care and self-management capabilities [26]. Help-seeking is made up of different components, including: knowledge about help-seeking and appropriate sources of support, attitudes towards help-seeking behaviours and mental health services, intentions to, or likelihood, of seeking help if needed, help-seeking confidence, mental health first aid, help-seeking stigma, perceptions around barriers, sources of support as well as treatments, and actual help-seeking behaviours [29, 31]. Each of these components can be intrapersonal, i.e., for self (e.g., self-stigma for seeking help or intentions to seek help for self), or interpersonal, i.e., for others (e.g., mental health first aid knowledge—knowing how to help others experiencing mental health difficulties or confidence helping a friend). There is also a distinction between formal and informal help-seeking in terms of sources of support (e.g., seeing a doctor vs. asking a teacher/parent/friend for help). However, few studies of MHL interventions focus on all components of help-seeking efficacy, instead selecting a single element as the outcome of interest [29].

For instance, regarding interpersonal help-seeking, mental health first aid interventions are often used to promote confidence in helping others. They typically do this by increasing knowledge of different mental health difficulties and available sources of support. An evaluation of ‘Teen Mental Health First Aid’ demonstrated that the intervention led to moderate short term increases in confidence and helpful intentions towards sample vignettes about suicide and anxiety [32]. Similarly, the ‘Finding Space for Mental Health’ efficacy trial, found that compared to a control group, CYP who received the intervention were significantly more likely to provide help to others who presented with mental health difficulties, with females demonstrating greater gains in first aid skills than males. In addition, participants in the intervention group were more likely to engage in help-seeking themselves [33].

In terms of CYPs actual help-seeking behaviours, several studies have identified significant increases in self-help-seeking behaviours among CYP in the months following receipt of a MHL intervention. For instance, a randomised controlled trial of the ‘Adolescent Depression Awareness Program’ [34] found that participants reported significantly more help-seeking behaviour, as well as an increase in treatment receipt, at the four-month follow-up stage, regardless of past medical history or family history of depression. Second, ‘Building Bridges to General Practice’ [35] demonstrated significant reductions in perceived knowledge- and belief-based barriers to consulting a General Practitioner (GP) in the intervention group, relative to the control classes. There were also significant increases in help-seeking intentions for psychological problems, and a significant association between participants’ intentions to seek help and their actual help-seeking behaviours (measured by subsequent self-reported GP consultations).

As actual help-seeking behaviours cannot always be identified immediately following delivery of an intervention, studies often look at intended help-seeking. For instance, Conrad et al. [36] conducted a quasi-experimental study of a three-part German school-based programme, ‘Crazy? So what!’ to promote mental health and reduce stigma. Results indicated that following implementation of the programme, pupils were significantly more likely to talk to their teacher about mental health difficulties (5.2% vs. 10.6%). They were also more likely to have positive attitudes towards others experiencing mental health difficulties, although this effect was not sustained over time. Conversely, Rickwood et al. [37] explored the impact of the ‘Mental Illness Education programme’ on intended help-seeking, as well as knowledge and stigma. While the programme had a strong impact on the latter two outcomes, there was only a weak impact on changing help-seeking intentions. However, unlike Conrad et al. [36], this study examined help-seeking from formal as well as informal sources which may explain the discrepancy in findings.

Despite the availability of the examples used above to illustrate content of such interventions, there is a lack of systematic investigations of the impact of universal, school-based interventions on help-seeking efficacy, investigating its different components (e.g. intended help seeking and attitudes) in CYP.

### What makes a help-seeking intervention successful?

Interventions have taken different stances in what they believe to be important in increasing help seeking and MHL. For example, underpinned by a behaviour change methodology, ‘Teen Mental Health First Aid’ posits that addressing barriers such as knowledge of mental health warning signs and support services, as well as how to talk about mental health, are core components [38]. Conversely, ‘Crazy? So what!’ asserts that contact with those who have lived experience positively affects knowledge, resulting in more positive attitudes towards mental health [36].

In terms of key components which may contribute to the development of a ‘successful’ intervention, a few factors seem promising. The Theory of Planned Behaviour [TPB; 39] is the most widely used theory in help-seeking interventions [40]. According to the TPB, intention is the proximal cause of behaviour, whilst beliefs (behavioural, normative and perceived behavioural control) are the most important determinants of intention. However, it is posited that perceived behavioural control can also directly predict and/or moderate the relationship between intention and behaviour. There is evidence for the effectiveness of the TPB model of help-seeking, with Webb and Sheeran’s [40] meta-analysis of 47 experimental health intervention trials finding that “interventions that produced greater intentions change had a corresponding greater effect on behaviour” (p. 256). Other general components which may contribute to successful interventions include making sure content is easy to understand and does not overload participants [41], whilst more specific components may depend on the aims and target populations. For example, males may benefit from the use of role-models, psychoeducational material to improve mental health knowledge, and sign posting to services [42].

### Methodological limitations in the literature

The issue identified above, whereby results vary according to the source of support being investigated, is one of several concerns currently prevalent in the help-seeking literature. Few studies comprehensively assess help-seeking efficacy from a range of reporters, with many focussed only on help-seeking intentions and not behaviours [29]. Additionally, reviews contributing to the topic [43–50] tend to focus on MHL as a broader topic with help-seeking efficacy not being split down into intra- or interpersonal, therefore, conflating findings which may yield different outcomes. A further important consideration is that often studies only report statistical significance, rather than effect size. Statistical significance does not inform about the magnitude of an effect and limits the ability to draw conclusions about practical relevance [51]. Effect sizes, especially when aggregated in a meta-analysis, may help distinguishing between programmes in terms of practical relevance [51]. Moreover, like other research exploring the effectiveness of school-based interventions [52], little is known about the sustained effects of help-seeking efficacy interventions [53].

### The current study

In light of the issues raised above, the current study aims to investigate the impact of universal, school-based interventions on help-seeking efficacy, investigating its different components (e.g. intended help seeking and attitudes) in CYP. Specific objectives are to:

(i) Describe this impact (measured as an effect size) at initial follow-up;
(ii) Describe this impact (effect size) at longer term follow-up(s).

## Method

In PROSPERO, a protocol was published on 12th May 2020; ID CRD42020188882]. The method presented below follows the relevant PRISMA reporting guidelines for systematic literature reviews [54].

### Eligibility criteria

To be eligible, studies were required to meet the following criteria relating to the participants, interventions, comparators, and outcomes (PICO). Studies with child and adolescent samples aged eight to 18 years were included as this would encapsulate both primary and secondary school pupils, who were able to self-complete measures on help-seeking constructs. If the mean sample age fell outside of this range, the study was removed. Any quantitative evaluations of universal, school-based interventions, with the aim of improving child and adolescent help-seeking efficacy, were eligible for inclusion. Studies conducted outside of the school setting were excluded. A control comparator (e.g. randomised or comparison to a historical control) was required.

In line with previous reviews of the literature [29, 43] studies were included if they measured any of the following dimensions of help-seeking: knowledge, stigma, confidence, intentions and behaviours, perceived help-seeking barriers and perceived helpfulness of referrals, help-sources and treatments, and mental health first aid intentions and behaviours for supporting others. Given the focus on intervention impact, an intervention effect size was also required (or data that could be used to calculate an effect size) that quantified a cross-sectional, group level difference between the intervention and control conditions on a help-seeking outcome.

Only studies available in English were eligible. Universal prevention programmes with a focus on general mental health and specific diagnoses (e.g., helping yourself or someone else experiencing depression) were included, however, interventions that were targeted only to specific child and adolescent populations e.g., those identified as experiencing depression, were excluded. Interventions with a primary focus on substance abuse were also excluded given the very large body of literature relating to risky health behaviours, which despite overlapping with the mental health help-seeking literature, was not within the scope of the current review. Previous reviews have taken this approach [55]. Interventions that were targeted to specific child and adolescent populations, such as programmes for those who were already depressed were also excluded.

### Search strategy

A search strategy was developed to map onto the PICO criteria. Child and adolescent populations were captured using terms such as ‘adolescen*’, ‘child*’ ‘teen*’ and ‘pupil*’, as well as terms relating to the school context. Intervention terms were included to capture the range of possible programmes targeting help-seeking outcomes e.g. ‘curricul*’, ‘psychoeducat*’ and ‘literacy’. To limit the search to studies with control comparators, design terms were used such as ‘random*’ and ‘control*’. Finally, to identify studies with relevant outcomes, help-seeking terms such as ‘help seeking*’ and ‘help-seeking*’ were included. For a full example search strategy, see Supplementary File 1 ‘Concepts and Search Strategy’.

### Data sources

The Cochrane Database of Systematic Reviews was searched to identify existing reviews relevant to the current study, and the reference sections were screened for eligible studies. In addition, the following databases were searched from their start date (1846 PsychInfo; 1946 Medline; 1974 Embase) until 21st May 2020. Experts in the field were also contacted for additional studies and to check that the search had captured expected studies.

### Study selection

**Fig. 1:**
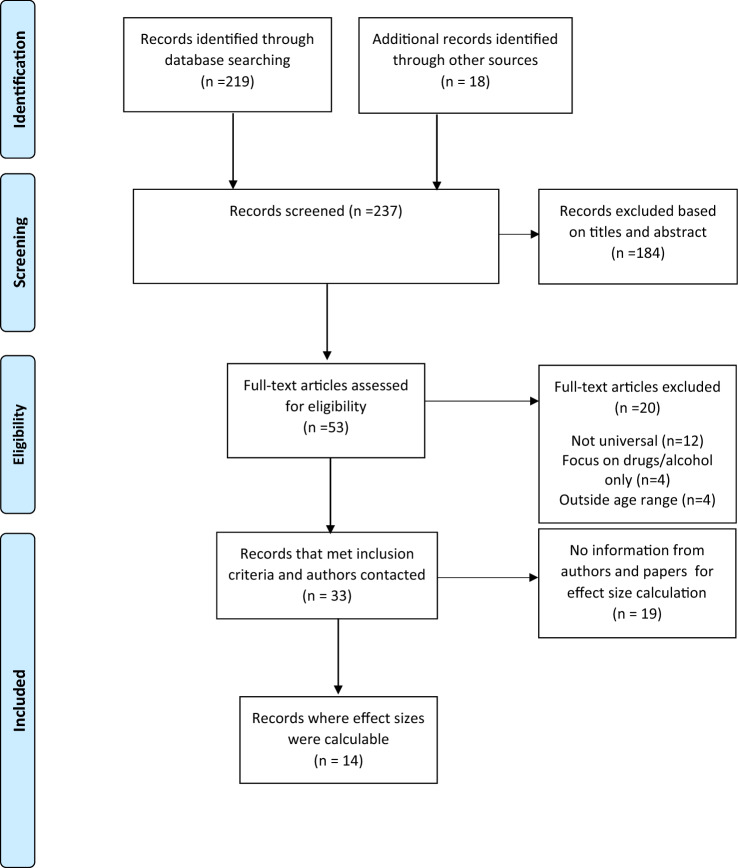
PRISMA flow diagram of study selection (adapted from Moher et al., 2009)

The results from the database searches were extracted and combined in Excel and duplicates were removed. Studies were screened and selected in two stages. In the first stage, the lead (DH), second (RM) and third (CM) authors each independently screened all the title and abstracts against the eligibility criteria, and then came together to review any uncertainties. For the second stage, the first (DH) and third (CM) authors each independently read and screened each full-text study to identify studies for inclusion. For cases where there was uncertainty about a study, the second author (RM) was consulted to reach a consensus. The flow of information from searching databases through to final study inclusion is outlined in Fig. 1.

### Data extraction

Data were extracted from eligible studies by the first (DH) and forth author (JS). A uniform data extraction form was created to record the following methodological information and results: study aim, country of origin, design, participant, school, and intervention characteristics, the help-seeking concept measured (e.g. knowledge, attitude/intention, behaviour) and data collection methods used (e.g. online vs. paper-based), the duration between baseline and each follow-up (if applicable) and effect sizes at each follow-up. The use of theory was subsequently extracted based on expert advice.

### Data synthesis and analysis

The methodology of included studies was synthesised and the proportion of studies with certain characteristics (e.g. Randomised control trial (RCT) design) summarised. When available, Cohen's d was calculated for any help-seeking efficacy dimensions, indicating the group level difference between the intervention and control conditions. For those studies that did not provide this information, authors were contacted and, where possible, Cohen’s d calculated based on the data made available. Due to the small number of studies included in the final sample, which explored different help seeking efficacy dimensions at different timepoints, a systematic narrative synthesis [56] was conducted. This involved the following steps: (a) exploring and organising the data to explore the direction and size of effects, (b) considering any factors that explain differences and (c) drawing conclusions around the strength of the evidence.

### Risk of bias (Quality) assessment

In addition to a summary of study methods and results, all studies were quality assessed using the Effective Public Health Practice Project (EPHPP) Quality Assessment Method which is acceptable for examining both randomised and non-randomised studies [57].

This enabled an assessment of bias within studies on the following domains: selection bias, study design, confounding variables, blinding, data collection methods, and withdrawal and drop out. A summary of risk of bias is presented for each study in Table 1 alongside the methodological characteristics and results.

**Table 1:** EPHPP quality assessment method for risk of bias

Paper	Selection Bias	Study design	Confounders	Blinding	Data Collection	Withdrawal and Dropout	Overall
Aseltine (2004)	Moderate	Strong	Strong	Weak	Weak	Strong	Weak
Aseltine (2007)	Moderate	Strong	Strong	Weak	Weak	Strong	Weak
Campos (2018)	Moderate	Strong	Strong	Weak	Strong	Moderate	Moderate
Chisholm (2016)	Strong	Strong	Moderate	Weak	Weak	Strong	Weak
Esters (1998)	Moderate	Moderate	Weak	Weak	Moderate	Strong	Weak
Fitzpatrick (2013)	Moderate	Strong	Strong	Weak	Weak	Moderate	Weak
Howard (2018)	Moderate	Strong	Moderate	Strong	Strong	Strong	Strong
Lai (2016)	Moderate	Strong	Strong	Weak	Weak	Moderate	Weak
Perry (2014)	Moderate	Strong	Strong	Strong	Strong	Weak	Moderate
Ravindran (2018)	Weak	Moderate	Strong	Weak	Strong	Moderate	Weak
Ruble (2013)	Moderate	Moderate	Weak	Weak	Strong	Weak	Weak
Strunk (2014)	Strong	Moderate	Moderate	Weak	Weak	Strong	Weak
Wong (2012)	Moderate	Moderate	Weak	Weak	Strong	Moderate	Weak
Wymen (2010)	Moderate	Strong	Strong	Weak	Weak	N/A	Weak

## Results

Database searching yielded 219 studies, whilst consultation with experts and handsearching references yielded two and 16 studies, respectively. The screening of titles and abstracts (first stage screening) resulted in the exclusion of 184 studies. Next, full-text screening (second stage screening) resulted in the exclusion of 20 studies. This resulted in 33 studies meeting inclusion criteria prior to effect sizes being calculated. Further information was requested from 20 study authors to be able to calculate effect size, five responded with 4 saying further information was not available and 1 providing further information.

### Studies that met inclusion criteria

Fourteen studies met the inclusion criteria for the review. Study characteristics are summarised in Table 2. Six originated from the United States of America (USA) [58–63], two from Australia [64, 65], two from Hong Kong [66, 67], and one from each of the following countries: the UK [68], Nicaragua [69], Ireland [70] and Portugal [33]. All interventions took place in secondary schools.

**Table 2:** Characteristics of studies examining help seeking dimensions

	Author and country	Design and comparator(s)	Intervention and theoretical underpinning	Sample characteristics^#^	Interpersonal or intrapersonal	Help-seeking measure(s) used	Number of timepoints	Effect size at each timepoint (Cohens *d*)
**Intended help seeking**								
1	Fitzpatrick et al., (2013). Ireland	2 arm RCT (enhanced intervention versus standard lessons)	‘Working Things Out’ nine stories over 12, 40 minute classes to help bring the mental health promotion aspects of the standard modules to life Theory not specified	Sample size: Total 782 Control 421 Intervention 361 Overall Age: M 13.6 (SD 0.59) Gender 53% male	Intrapersonal	Self-made measure	2	End of intervention − 0.06 months later − 0.03
2	Howard et al., (2018). Australia	3 arm RCT (2 active arms: biological and psychological perspectives on mental health) versus no intervention	30-minute online video outlining a) biological causes of depression or b) psychological causes of depression Theory mentioned, but not specified	Sample size: Total 327 Control 114 (49% male). Age Mdn 16 Intervention (B) 122 (48% male). Age Mdn 16 Intervention (P) 91 (46% male). Age Mdn 17 Overall Gender 48% male Age Mdn 17	Intrapersonal	General Help-seekingQuestionnaire (GHSQ; Wilson, Deane, Ciarrochi, & Rickwood, 2005)	1	End of intervention Biological arm vs control 0.24 Psychological arm vs control 0.28
3	Lai et al. (2016). China	3 Quasi-experimental design (2 active arms: Professional and teacher led courses of the Little Prince ins Depressed) versus no intervention	12 session course ‘The Little Prince is depressed’ Theory mentioned, but not specified	Sample size: Total 2304 Control 698 (65% male). Age M 15.3 (SD 1.0) Intervention (PL) 943 (47% male). Age M 15.5 (SD 1.0) Intervention (TL) 663 (42% male). Age M 14.5 (SD 0.8) Overall Gender 51% male Age M 15.2 (SD 0.9)	Intrapersonal	Self-made measure	2	End of intervention Professional led:Prof: – 0.02 Family: 0.08 Friend: 0.13 Teacher: – 0.04 Teacher led: Prof: – 0.08 Family: 0.08 Friend: 0.09 Teacher: – 0.12 4-5 months later Professional led: Prof: – 0.07 Family: 0.05 Friend: 0.17 Teacher: – 0.09 Teacher led:Prof: 0.00 Family: 0.12 Friend: 0.15 Teacher: 0.00
4	Rivindran et al., (2018). Nicaragua	2 arm RCT (intervention versus no intervention)	12-week course ‘The Guide’ Theory not specified	Sample size: Total 620 Control 214 (34% male^). Age^ M 18.1 (SD 2.0) Intervention 406 (44% male^). Age^ M 17.6 (SD 2.0) Overall Gender 41% Age M 17.8 (SD 2.0)	Intrapersonal	GHSQ (Wilson, Deane, Ciarrochi, & Rickwood, 2005).	1	End of intervention 0.22
5	Wyman et al., (2010). USA	2 arm RCT (intervention versus no intervention)	15 module course ‘Sources of Strength’ for suicide prevention Underpinned by Diffusion of Innovations Theory	Sample size: Total 2675 Control 918 (48% male) Intervention 1757 (48% male) Overall Gender 48% male Age data not provided	Interpersonal	Self-made measure	1	End of intervention Friend to get help from an adult: 0.55
6	Strunk et al., (2014). USA	2 arm pre-post study (intervention versus no intervention)	4-day suicide prevention programme Underpinned by Social Cognitive Theory	Sample size: Total 1547 Control 581 (51% male). Age Mdn 15 Intervention 966 (56% male). Age Mdn 15 Overall Gender 54% male Age Mdn 15	Both (intra and interpersonal)	Self-made measure	1	End of intervention Help friend or self 0.12
**Attitude towards help seeking**								
1	Chisholm et al., 2016). UK	2 arm RCT (contact and education intervention versus education only)	One day, ten module programme Underpinned by Intergroup Contact Theory	Sample size: Total 657 Control 303 (48% male) Intervention 354 (58% male) Overall Gender 53% male Age^ M 12.2 (SD 0.6)	Intrapersonal	Self-made measure	2	2 weeks post intervention 0.02 6 months later 0.04
2	Esters et al., (1998). USA	2 arm pre-post study (intervention versus no intervention)	270-minute course, spread over 1 week Theory not specified	Sample size: Total 40 Control 20 Intervention 20 Overall Age M 14.7 (SD not reported) Gender 34% male	Intrapersonal	Fisher-Turner ProCon Attitudes Scale (Fisher & Turner, 1970)	2	End of intervention 0.53 3 month later 0.59
3	Perry et al., (2013). Australia	2 arm RCT (intervention versus no intervention)	12 hours of lessons, delivered over 5-8 weeks Theory not specified	Sample size: Total (end of intervention) 204 Control 153 Intervention 159 Total (6 months later) 204 Control67 Intervention137 Overall Age^ M 14.8 (SD not reported) Gender^ 50% male	Intrapersonal	Inventory of Attitudes towards Seeking mental Health Services (Fisher & Turner, 1970)	2	End of intervention 0.05 6 months later − 0.02
4	Wong et al., (2012). China	3 Quasi-experimental design (2 active arms: Professional and teacher led) versus no intervention	12 session course 'The Little Prince is depressed' Theory not specified	Sample size: Total 280 Control 120 (not specified) Intervention* 160 (67% male) Overall Gender (not specified) Age 14-16	Intrapersonal	Attitudes Toward Seeking Professional Psychological Help: AShortened Form (ATSPPH-SF; Fischer & Farina, 1995)	1	End of intervention 0.13
5	Wyman et al., (2010). USA	2 arm RCT (intervention versus no intervention)	15 module course ‘Sources of Strength’ for suicide prevention Underpinned by Diffusion of Innovations Theory	Sample size: Total 2675 Control 918 (48% male) Intervention 1757 (48% male) Overall Gender 47% Age data not provided	Interpersonal	Self-made measure	1	End of intervention 0.63
6	Ruble et al., (2013). USA	2 arm pre-post study (intervention versus no intervention)	3-hour depression literacy programme Theory not specified	Sample size: Total 475 Control 143 Intervention 332 Gender 38% male Age 14-15	Interpersonal	ADAP Depression Knowledge Questionnaire (ADKQ; Hart et al., 2014)).	1	6 weeks later 0.25
7	Aseltine et al., (2007). USA	2 arm RCT (intervention versus no intervention)	2-day programme on suicide prevention Theory not specified	Sample size: Total 4133 Control 2094 Intervention 2039 Gender ^49% male Age^ Mdn 14-15 category	Interpersonal	Self-made measure	1	3 months post intervention 0.38
8	Aseltine et al., (2004). USA	2 arm RCT (intervention versus no intervention)	2-day programme on suicide prevention Theory not specified	Sample size: Total 2100 Control 1073 Intervention 1027 Gender ^49% male Age^ Mdn 14-15 category	Interpersonal	Self-made measure	1	3 months post intervention 0.24
**Self-help strategies**								
1	Campos et al., (2018). Portgual	2 arm RCT (intervention versus no intervention)	2, 90-minute sessions, one week apart Theory not specified	Sample size: Total 502 (1 week post intervention) Control 263 Intervention 239 Total 307 (6 months later) Control 176 Intervention 211 Gender^ 52% male Age^ Mdn 13 category	Intrapersonal	Self-made measure	2	1 week post intervention 0.52 6 months later 0.04
**First aid skills and help seeking**								
1	Campos et al., (2018). Portgual	2 arm RCT (intervention versus no intervention)	2, 90 minute sessions, one week apart Theory not specified	Sample size: Total 502 (1 week post intervention) Control 263 Intervention 239 Total 307 (6 months later) Control 176 Intervention 211 Gender^ 52% male Age^ Mdn 13 category	Intrapersonal	Self-made measure	2	1 week post intervention 0.22 6 months later– 0.06
**Confidence in help seeking**								
1	Strunk et al., (2014). USA	2 arm pre-post study (intervention versus no intervention)	4 day suicide prevention programme Underpinned by Social Cognitive Theory	Sample size: Total 1547 Control 581 (51% male). Age Mdn 15 Intervention 966 (56% male). Age Mdn 15	Intrapersonal	Self-made measure	1	End of intervention 0.47
**Help-seeking stigma**								
1	Strunk et al., (2014). USA	2 arm pre-post study (intervention versus no intervention)	4 day suicide prevention programme Underpinned by Social Cognitive Theory	Sample size: Total 1547 Control 581 (51% male). Age Mdn 15 Intervention 966 (56% male). Age Mdn 15	Intrapersonal	Self-made measure	1	End of intervention 0.27
**Actual help seeking**								
1	Aseltine et al., (2007). USA	2 arm RCT (intervention versus no intervention)	2-day programme on suicide prevention Theory not specified	Sample size: Total 4133 Control 2094 Intervention 2039 Gender ^49% male Age^ Mdn 14-15 category	Interpersonal	Self-made measure	**1**	3 months post intervention 0.10
2	Aseltine et al., (2004). USA	2 arm RCT (intervention versus no intervention)	2-day programme on suicide prevention Theory not specified	Sample size: Total 2100 Control 1073 Intervention 1027 Gender ^49% male Age^ Mdn 14-15 category	Interpersonal	Self-made measure	**1**	3 months post intervention 0.22

# When available, data is broken down into intervention arm, age and gender, *Intervention arms analysed together in study, B = Biological arm, P = Psychological arm, PL = Professional Lead, TL = Teacher Led, ^Reported only in baseline sample

Studies ranged from having between 40 [60] and 4133 young people [58]. Overall, the average age of young people in the study was between 12.2 [68] and 17.8 years [69]. Most studies were close to having a 50% split (±5%) across gender [33, 58, 59, 61, 62, 64–66, 68, 70], however, some were skewed towards having more female [60, 63, 69] participants and one did not specify the gender split [67]

Intervention intensity ranged from a single, 30-min video session [64] to a four-day suicide prevention programme [61]. Four interventions were delivered by a health professional [33, 61, 63, 68], five were delivered by teachers or other school staff who did not have a health background [58, 59, 65, 69, 70], in one the facilitator/deliverers role was unclear [60], one by both professionals and school staff [67], one online [64], one by both staff and pupils [62], and one by either a healthcare professional or teacher depending on the intervention arm students were allocated to [66]. Theory, or theoretical underpinning, was explained/reported/detailed in five studies [61, 62, 64, 66, 68]. Two studies mentioned non-specific underpinnings and three referred to a specific theory: Diffusions of Innovation Theory [71], Social Cognitive Theory [72] and Intergroup Contact Theory [73]. These are further outlined in Table 2.

Intended help seeking was explored in six studies [61, 62, 66, 70, 74]. Help-seeking attitudes were explored in eight studies [58–60, 62, 63, 65, 67, 68]. Actual help seeking behaviour was explored in two studies, where the second study was an expansion of the first including data from the original study [58, 59]. Other relevant constructs related to help seeking included: intentions for self-help strategies [33], help seeking confidence [61], help seeking stigma [61], and a combined construct of mental health first aid skills and intended help seeking [33]. Outcomes could also be further delineated into intrapersonal i.e., for self (e.g., self-stigma for seeking help or intentions to seek help for self) and interpersonal i.e., for others (e.g., mental health first aid knowledge—knowing how to help others experiencing mental health difficulties or confidence helping a friend). [29].

### Quality assessment of included studies

The results from the EPHPP quality assessment are depicted in Table 1. Of the fourteen studies, one [64] was rated strong overall (as indicated by no weak ratings across any of the EPHPP criteria). Two [33, 65] were rated as ‘moderate’ overall (as indicated by one weak rating across all quality assessment criteria) and ten were rated as ‘weak’ overall (as indicated by two or more ‘weak’ ratings across all quality assessment criteria). The categories ‘study design’ and ‘withdrawal and drop out’ received the highest frequency of strong ratings, whilst ‘making sure outcome assessors were blinded’ and ‘data collection methods’ received the highest frequency of weak ratings. As most studies were rated weak, findings, detailed below, should be treated cautiously.

### Studies presented in this review

Given the multitude of potential components relevant to help seeking, results below focus on those which were examined across more than one study and were not only an expansion of a previous study drawing on the same data: i) intrapersonal intended help-seeking, ii) intrapersonal attitudes towards help seeking, and iii) interpersonal attitudes towards help seeking. An overview of other constructs, including; interpersonal intended help seeking, confidence in help-seeking, help seeking stigma, behavioural intentions for self-help strategies, and first aid skills and help seeking are outlined in the supplementary information.

#### Impact of programmes on intended help-seeking

Four studies where effect sizes could be calculated looked at intrapersonal (self) intended help seeking [64, 66, 69, 70].

#### Intrapersonal intended help seeking

##### Initial post intervention follow-up

All four studies reported on intrapersonal intended help seeking at the conclusion of the intervention [64, 66, 69, 70]. One study explored whether there would be a difference in outcomes depending on whether students were educated on the underlying causes of depression using a 30-min video exploring either biological or psychological perspectives, which were then compared to those who received no intervention [64]. At the end of the video, an effect size of 0.24 was demonstrated on intended help seeking in the biological arm and 0.28 for the psychological arm. A longer 12-week mental health curriculum intervention, referred to as ‘The Guide’ was piloted in Nicaraguan secondary schools [69]. Delivered by school staff, with the aim of improving mental health literacy, students were allocated to receive ‘The Guide’ or no intervention. At the end of the programme, an effect size of 0.22 was found on intended help seeking. ‘Working Things Out’ was another 12-session intervention, but aimed specifically at mental health promotion [70]. This took place over eight months in Ireland and guided pupils through different mental health scenarios via videos and stories. Discussion and other exercises were then facilitated by a teacher. A negative effect size was found at the end of the programme (− 0.06). Lastly, an intervention called ‘The Little Prince is Depressed’ consisted of another 12-session programme spread over 4–5 months [66]. The programme's aim was to prevent depression in pupils based in Hong Kong. Pupils were allocated to receive the intervention: (a) by their teacher, (b) by a healthcare professional; or to receive no intervention. Help-seeking was measured by exploring the likelihood pupils would seek help from a professional, family member, friend, or teacher. At the end of the intervention, effect sizes of between -0.08 and 0.13 were demonstrated on intended hep seeking, depending on the source.

##### Longer term follow-up

Two studies, ‘The Little Prince is Depressed’ [66] and ‘Working Things Out’ [70] completed a longer term follow-up once the intervention had ceased. One study had a follow-up four to five months later [66], whilst the other had a follow-up at six months [70]. At the four to five month follow-up for the ‘The Little Prince is Depressed’ [66] effect sizes of between − 0.09 and 0.17 were found on intended help seeking, depending on source. Conversely, for ‘Working Things Out’ [70] a negative effect size was demonstrated at the six month follow-up for intended help seeking (-0.30).

### Impact of programmes on attitudes towards help-seeking

Of the eight studies where effect sizes could be calculated and which explored attitudes towards help seeking, four explored intrapersonal attitudes [60, 65, 67, 68] and four explored interpersonal attitudes [58, 59, 62, 63].

#### Intrapersonal attitudes towards help seeking

##### Initial post intervention follow-up

All four studies reported an outcome of intrapersonal attitudes towards help seeking at the end of the intervention [60, 65, 67, 68]. The ‘SchoolSpace’ programme [68] was a one day-10 module syllabus which aimed to improve mental health literacy. Students were either allocated to an educational programme about mental health delivered by a mental health professional, or to an enhanced programme with the educational component plus contact with an individual who previously had a mental health difficulty. At the end of the intervention an effect size of 0.02 was found when comparing the active control (education only) to the intervention (education plus contact with a service user). Another unnamed programme aimed to raise awareness and change attitudes towards individuals with mental health difficulties [60]. This was via a 270-min classroom-based intervention spread over 1 week. Students were allocated to the intervention or the control group. At the end of the programme, an effect size of 0.53 was demonstrated for students’ attitudes towards help seeking. ‘Headstrong’ [65] aimed to improve mental health literacy in secondary school pupils in Australia. In total, the programme consisted of 12 h of content, delivered by a teacher over 5–8 weeks. Students were either allocated to ‘Headstrong’ or a control group. At the end of the intervention, an effect size of 0.05 was demonstrated. Lastly, in ‘The Little Prince is Depressed’ [66], an effect size of 0.13 was found for intrapersonal attitudes towards help seeking.

##### Longer term follow-up

Three of the five studies completed longer term follow-up on intrapersonal attitudes towards help seeking. Follow up ranged from three [60] to six months [65, 68]. In the study which aimed to change students' awareness and perceptions of those with mental health difficulties [60], a medium effect size of 0.59 was maintained at three months post intervention. At six months follow-up, the Headstrong intervention [65] demonstrated a similar effect size to that obtained immediately after the intervention (0.02 at six months to 0.05 after the intervention). The SchoolSpace intervention [68] only followed up two schools (33% of the total involved in the project) at six month follow-up. A similar effect size was obtained both after the intervention and at the six month follow-up (0.04 at six month follow-up versus 0.02 after the intervention).

#### Interpersonal attitudes towards help seeking

##### Initial post intervention follow-up

The ‘Sources of Strength’ suicide prevention programme [62] was a 3-month, 3 phase intervention aimed at preventing suicide in the USA. Delivered by both staff and pupils, students were allocated to receive the intervention or to a control condition. At the end of the intervention an effect size of 0.63 was found on perceptions of adult help for suicidal peers.

##### Longer term follow-up

Three studies explored the short-term effect of an intervention on interpersonal attitudes at follow-up. One explored the effects six weeks later [63], whilst the other two studies explored this at three months [58, 59]. The Adolescent Depression Awareness Program [63] was a three-hour depression literacy programme. This was aimed at secondary students and taught by individuals with a medical background. Students were either allocated to a control group or the Adolescent Depression Awareness Program. At six-week follow-up an effect size of 0.25 was obtained for interpersonal attitudes towards help seeking. Two other studies, conducted by the same author, explored the ‘SOS Suicide Prevention Programme’ in secondary school students in the USA [58, 59]. This consisted of two components: one using videos and exploring suicide and depression; the other using a screening questionnaire for depression and suicidality. In the first study, an effect size of 0.38 was found for interpersonal attitudes towards help seeking. In the subsequent study, a replication and extension of the original study, a smaller effect size of 0.24 was found.

## Discussion

The aim of this review was to investigate the impact of universal, school-based interventions on help-seeking in children and young people, as well as to explore longer term impact. Whilst there was a diverse mix of both interventions and constructs explored, three constructs were examined across multiple studies: (a) intrapersonal attitudes towards help-seeking, (b) interpersonal attitudes towards help-seeking and (c) intrapersonal intended help-seeking.

For intrapersonal attitudes towards help-seeking at the end of the intervention, one study found a medium effect size [60], whilst three found negligible effect sizes [65, 67, 68]. No differences on aspects such as age or the use of theory could be identified. However, aspects relating to gender or intervention length may account for any differences. The study with a medium effect size has a lower percentage of males taking part [60]. As males are less likely to engage in help seeking [10], this could account for this difference. Alternatively, the intervention length, of short bursts over 1 week may have helped individuals engage and not feel overloaded, resulting in information being processed and encoded by the young people [41]. Longer term follow-up ranged from three to six months and was recorded in three studies. However, it appears that from the limited evidence available, effect sizes remained relatively stable to post intervention measurement.

Four studies explored interpersonal attitudes towards help-seeking. However, only one recorded this outcome at the end of the intervention, where a medium effect size was found [62]. Three studies recorded longer term post intervention follow ups, but did not measure at the end of the intervention, for each, small effect sizes were observed; 0.25 in the Adolescent Depression Awareness Program [63], 0.34 in the original SOS programme [59] and 0.24 in the larger SOS replication study [58]. Commonalities across all studies producing small to medium effect sizes were the age range with all participants being 15–16 and a focus on diagnosis specific knowledge content. The study which demonstrated a medium effect size was longer in duration and underpinned by theory [62] suggesting these could be important considerations for the effectiveness of interventions on interpersonal attitudes towards help seeking. The TPB [39] has been found as an important factor in help seeking interventions [40], yet this intervention utilised the Diffusions of Innovation Theory [71], which seeks to explain how, why, and at what rate new ideas (in this case pertaining to suicide) spread. Identifying and measuring the specific change mechanisms underpinning this theory will allow for a better understanding of how the intervention effects interpersonal attitudes towards help-seeking. In terms of sustained effects, there was no longer term follow-up on this study meaning it is impossible to say whether the medium effect size was limited to when measurement was taken.

Four studies explored intrapersonal intended help seeking at the end of the intervention. Two studies reported small effect sizes [64, 74], whilst two reported negligible effect size [66, 70]. For studies which demonstrated small effect sizes, young people were older adolescents (aged 17). Targeting interventions at this age may be effective as young people are gaining independence and are not as reliant as younger individuals on others, such as parents and guardians, to seek help [75]. Similar to constructs discussed above, there does not appear to be any obvious differences regarding intervention length or deliverer. For example, ‘The Guide’ [74] was delivered by teachers over 12, 60-min sessions and a small effect size was demonstrated, whilst ‘the Little Prince is Depressed’ [66] was similar in terms of length, but had a negligible effect size at follow-up for both teacher and professional arms. Longer term follow-up ranged from four-to-six months. Similar to effect sizes at the end of the intervention, negligible effect sizes were found at four month follow-up for ‘the Little Prince is Depressed’ [66]. Interestingly, ‘Working Things Out’ [70] demonstrated a small negative effect size six months after the intervention; indicating that those in the control group stated they were more likely to seek help than those in the intervention group. Differences in peer problems in the control and intervention groups, with the intervention group scoring higher at baseline, could account for some of these differences, indicating a child that does not interact well with others may not benefit from help-seeking interventions.

### Methodological issues in the field

In terms of data collection methods, it is widely acknowledged that there is improvement needed around measurement and help-seeking efficacy, with studies either using single item measures designed by study authors, or using general measures where psychometric properties are unclear [29]. Without robust and validated measures for this age group, it is impossible to be able to ascertain with any degree of certainty the impact that the interventions being evaluated have had on CYP’s help-seeking efficacy. The development of validated measurement tools is vital if the data are to be accurately collected and the outcomes appropriately evaluated.

Further attention needs to be paid to implementation factors around MHL and help seeking interventions [29]. When conducting efficacy studies, few school-based MHL programmes include implementation metrics, and those that do, often focus on one metric (e.g. fidelity) [68]. To be able to understand successful interventions, a greater understanding of the interplay between implementation and efficacy is needed, with both qualitative and quantitative methods being drawn on to provide a comprehensive account on what works for who, and under what circumstances.

For longer term, post-intervention follow-up, nine interventions had calculable effect sizes reported. Despite this, the longest follow-up was six months post intervention [33, 65, 70]. Long term follow-up is an issue in the field of school mental health research [53]. Most studies have short term (i.e., less than 6 months) follow-up only and the small number that do include longer term follow-up tend to show mixed or limited effects [76]. However, exploring whether effects are sustained over time is vital as the help-seeking skills and attitudes developed by CYP during the intervention are unlikely to be needed immediately by most participants, but may well be of use in the future when difficulties arise. Embedding mental health education into the curriculum may be one possible way of ‘topping up’ any demonstratable effect size from short help seeking interventions. Some countries are already implementing such methods [14, 77], but their long-term impact for interventions such as help-seeking has yet to be established. Additionally, it is also important to note that help-seeking intentions do not always translate into actual behaviour change [78] and the structural barriers to help-seeking must therefore also be accounted for [8, 79].

### Strengths and limitations

A strength of this study is the use of three researchers at first stage screening all of whom independently reviewed all studies at title and abstract stage (DH, RM, CM). Additionally, two researchers independently reviewed all studies at full text screening stage (DM, CH). Having all studies independently double-screened mitigates the risk of systematic bias. A similar approach was employed at data extraction stage and quality assessment by two researchers (DH and JS) which again, mitigates against bias and decreases the total number of errors in data extraction and quality assessment, with no differences being identified.

The main limitations pertinent to this study relate to the quality of the studies and their reporting. On the whole, the quality of studies identified in this review was weak, making it difficult to draw reliable conclusions about the impact of interventions reported. Furthermore, despite 33 studies meeting inclusion criteria, the information required to calculate effect sizes was only available for 14 of those (42%). It may be that findings would be different if more information was available, and therefore findings should be treated with caution, with only one study author able to provide further information to calculate the effect size. A further limitation is that this review only produced results pertaining to secondary schools, thus, the efficacy of help seeking interventions in primary schools remains unknown. Moreover, educational databases were not included in the search, thus, whilst reference searching and consultation with experts was employed, it is possible that some studies including those in primary schools, may have been missed. Lastly, fidelity to the intervention was only reported in four studies and therefore was not examined in this review. Without being able to explore in depth how interventions were delivered in practice, it is difficult to ascertain how these may impact effect size [80]

### Future research

Studies included in this review all occurred prior to the Covid-19 pandemic, due to the dates the search was undertaken. For many countries, the pandemic resulted in increased rates mental health difficulties in young people [81, 82], as well as disruption to schooling and education [83] and the ability to access services for mental health support [84], all of which are relevant to the topic in this review. Future studies exploring universal, school-based, help-seeking interventions, should account for these wider factors, along with other structural barriers, previously highlighted in the literature [8, 79].

In line with previous recommendations [29, 43], researchers working in the field of help-seeking should use existing valid and reliable measures to record outcomes. Intervention developers and researchers may also wish to draw on the use of logic models, which describes how the intervention meets the desired needs e.g., what core components are needed to achieve the outcome [85] and include these in supplementary materials. This will help crystalise thinking on what help seeking concepts the intervention is targeting and ensure that outcomes are appropriately operationalised. It will also help identify change mechanisms and moderators which should also be measured as part of any interventional study.

In addition to this, more methodologically robust studies are needed to advance the field. This includes having adequate sample sizes to ensure studies are appropriately powered, as well as making sure statisticians and outcome assessors are blind to intervention conditions and using valid and reliable measures. Research is also urgently needed to look at the longer term effects of help-seeking efficacy programmes for CYP. Ascertaining how long any potential effects last will, in turn, help schools and services to understand when ‘top ups’ may be needed to bolster gains made from previous programmes. Lastly, given the variability in effect sizes and that few overarching characteristics were linked to outcomes, exploring the makeup of the intervention in terms of behaviour change techniques, intervention functions, and fidelity to protocols, may help shed light on what constitutes an effective programme.

## Concluding remarks

Whilst help-seeking is outlined as being important in the early identification and treatment in child and youth mental health [3], evidence of effect sizes in relation to universal, school-based programmes is mixed, with the exception of interpersonal attitudes towards help seeking. Here, evidence from three studies suggests that small effect sizes are demonstrated for up to six months after the intervention has taken place. Additionally, what constitutes an effective programme to improve help seeking efficacy remains unclear; there often were no immediate differences in terms of intervention length, intensity, or deliverer, in effective versus ineffective programmes.

On the whole, the poor quality of studies and mixed findings summarised in this study do not give a clear endorsement of interventions to support help-seeking efficacy and do not provide good evidence of links between intended and actual help-seeking. Further work should be undertaken to (i) understand the core ingredients needed to deliver effective, universal school-based interventions to improve different help seeking constructs, (ii) address the structural barriers for mental health support seeking, (iii) explore implementation factors in help-seeking interventions and (iv) explore whether embedding mental health education more broadly across the school curriculum could top up small effect sizes preliminary gained from interventions explored in this review.

## Supplementary Information

Below is the link to the electronic supplementary material.

Supplementary file1 (DOCX 16 KB)
