# Supplementary material for: The impact of universal, school based, interventions on help seeking in children and young people: a systematic literature review
Source: Eur Child Adolesc Psychiatry. 2023 Jan 13;33(9):2911–28. doi: 10.1007/s00787-022-02135-y (PMC9837763; doi:10.1007/s00787-022-02135-y)
Supplement: Supplementary file 1 — Supplementary file1 (DOCX 16 KB) [file 787_2022_2135_MOESM1_ESM.docx]

1. **Impact of programmes on Interpersonal intended help seeking**

‘Sources of Strength’ [62] was a 3-month, 3 phase intervention aimed at preventing suicide in the USA. Delivered by both staff and pupils, students were allocated to receive the intervention or to a control condition. At the end of the intervention, the effect size for students reporting norms about getting help from adults was 0.55.

1. **Impact of programmes on Intended help-seeking (intrapersonal and interpersonal construct)**

A specific question in one study combined these two constructs, which meant these could not be disaggregated. Surviving the teens’ [61] was a four-day programme, delivered by a mental health professional in the USA. This was a both a suicide prevention and depression awareness programme, with students allocated to either the programme or to no intervention. At the end of the programme, an effect size of 0.12 was found.

1. **Impact of programmes on intrapersonal actual help seeking**

Two studies [58, 59] intrapersonal actual help seeking and only a post intervention follow up at three months. The content of the intervention is described above consisted of two components: one using videos and exploring suicide and depression; the other using a screening questionnaire for depression and suicidality. In the first published study [59], an effect size of 0.10 was found, whilst in the subsequent study replication on a larger scale [58], an effect size of 0.22 was found.

1. **Impact of programmes on other types of help seeking**

Four additional outcomes were found in studies which were related to constructs around help seeking. These included: ‘behavioural intentions for self-help’, as well as **‘**first aid skills and help seeking’, both in the Find Space for Mental Health Programme [32]. The further two constructs, ‘confidence in help seeking’ and ‘stigma around seeking help’ were evaluated in the Surviving the teens suicide prevention and depression awareness programme [61].

**3.1** **Impact of program on ‘behavioural intentions for self-help strategies’ as well as ‘first aid skills and help seeking’**

One study explored the impact of a mental health literacy programme on pupils’ behavioural intentions for self-help. The programme Find Space for Mental Health was conducted in Portuguese secondary schools [32]. It consisted of two, 90-minute sessions which were one week apart and explored mental health literacy via different videos, exercises and discussions. Sessions were facilitated by a Psychologist and a MSc Psychology student. This programme did not measure these outcomes straight after the intervention, but did explore them at 1 week follow up, as well as 6 months after the intervention. For ‘behavioural intentions for self-help strategies’ (intrapersonal), an effect size of 0.52 was found at 1-week post intervention, whilst at 6 months an effect size of 0.04 was found. For ‘first aid skills and help seeking’ (intrapersonal) at 1-week post intervention, an effect size of 0.22 was found, whilst six months later the effect size was -0.06.

**3.2 Impact of program on ‘confidence in help seeking’ and ‘help seeking stigma’**

The Surviving the Teens programme explored both ‘confidence in help seeking’ as well as ‘help seeking stigma’ [61]. This study only measured outcomes at the end of the intervention and did not look into longer term follow up. ‘Confidence towards help seeking’ was measured in two ways: confidence in helping a suicidal friend (interpersonal), as well as confidence in handling and talking about problems intrapersonal). For confidence in helping a suicidal friend, an effect size of 0.47 post intervention was obtained, whilst confidence in handling and talking about problems had an effect size of 0.05. For help-seeking stigma, the intervention resulted in an effect size of 0.27.
